# Family environment and adult resilience: contributions of positive parenting and the oxytocin receptor gene

**DOI:** 10.3402/ejpt.v4i0.21659

**Published:** 2013-09-18

**Authors:** Bekh Bradley, Telsie A. Davis, Aliza P. Wingo, Kristina B. Mercer, Kerry J. Ressler

**Affiliations:** 1 Atlanta VAMC, Department of Psychiatry and Behavioral Sciences, Emory University, Atlanta, GA, USA; 2 Department of Human Genetics, Howard Hughes Medical Institute, Emory University, Atlanta, GA, USA; 3 Department of Psychiatry and Behavioral Sciences, Howard Hughes Medical Institute, Yerkes Primate Research Center, Emory University, Atlanta, GA, USA

**Keywords:** Childhood maltreatment, abuse, family environment, resilience, positive affect, Connor–Davidson Resilience Scale (CD-RISC), oxytocin, OXTR, rs53576

## Abstract

**Background:**

Abundant research shows that childhood adversity increases the risk for adult psychopathology while research on influences of positive family environment on risk for psychopathology is limited. Similarly, a growing body of research examines genetic and gene by environment predictors of psychopathology, yet such research on predictors of resilience is sparse.

**Objectives:**

We examined the role of positive factors in childhood family environment (CFE) and the OXTR rs53576 genotype in predicting levels of adult resilient coping and positive affect. We also examined whether the relationship between positive factors in the CFEs and adult resilient coping and positive affect varied across OXTR rs53576 genotype.

**Methods:**

We gathered self-report data on childhood environment, trauma history, and adult resilience and positive affect in a sample of 971 African American adults.

**Results:**

We found that positive CFE was positively associated with higher levels of resilient coping and positive affect in adulthood after controlling for childhood maltreatment, other trauma, and symptoms of posttraumatic stress disorder. We did not find a direct effect of OXTR 53576 on a combined resilient coping/positive-affect-dependent variable, but we did find an interaction of OXTR rs53576 with family environment.

**Conclusions:**

Our data suggest that even in the face of adversity, positive aspects of the family environment may contribute to resilience. These results highlight the importance of considering protective developmental experiences and the interaction of such experiences with genetic variants in risk and resilience research.

Studies show a clear relationship between exposure to traumatic events and increased risk for physical and mental health problems across a lifespan (Anda et al., 2006). Child maltreatment is particularly associated with increased risk for mental health problems (McLaughlin, Conron, Koenen, & Gilman, 2010). Childhood maltreatment increases the likelihood for the development of stress and trauma-related psychological problems in adulthood (Cloitre et al., 2009). Although risk for psychological disorders is elevated following exposure to childhood maltreatment, mental or behavioral problems develop in only a minority of individuals with such a history (McGloin & Widom, 2001). While research on resilience following exposure to trauma and childhood maltreatment is growing (Feder, Nestler, & Charney, 2009), much of this research examines resilience as the absence of psychopathology or functional deficits: an approach that may overlook a number of important aspects of resilience (Walsh, Dawson, & Mattingly, 2010). Another approach to resilience focuses on adaptive coping styles and other personal attributes such as ego strength, tenacity, hardiness, optimism, self-efficacy, spirituality/faith, adaptive coping styles, and cognitive flexibility that appear to mitigate negative psychological sequelae in response to adversity (Feder et al., 2009). Positive affect is another attribute theorized to promote resilience via increasing flexible thinking, facilitating adaptive coping strategies, and counteracting the physiological effects of negative emotions (Fredrickson, 2001).

Only a few studies have examined genetic contributions to resilience (Boardman, Blalock, & Button, 2008; Kim-Cohen, Moffitt, Caspi, & Taylor, 2004). To date, we have found three published studies that examine relationships between candidate genes and resilience. One study (Stein, Campbell-Sills, & Gelernter, 2009) found that variation in the serotonin transporter promoter polymorphism (5HTTLPR) directly predicted the level of emotional resilience. Hankin and colleagues (Hankin et al., 2011) examined the interaction of 5HTTLPR and family environment as a predictor of positive affect in children and adolescents. They found that short (s) allele carriers appeared to be more genetically susceptible to the influence of parenting compared to long (l) allele carriers. Specifically, for individuals homozygous for the s allele, supportive parenting was associated with higher levels of positive affect and non-supportive parenting was associated with lower levels of positive affect. An additional manuscript reported that resilience was predicted by a gene × environment (G×E) interaction with child maltreatment and four genes associated with emotional responding (serotonin transporter, corticotropin-releasing hormone receptor 1, dopamine receptor D4, and oxytocin receptor) (Cicchetti & Rogosch, 2012). These studies, as well as other research (Hanson & Chen, 2010; Parade, Supple, & Helms, 2012), highlight the importance of considering the role of family environment in establishing a foundation for resilience in the face of trauma exposure (Gabalda, Thompson, & Kaslow, 2010). Variability in family environment exists even within families of maltreated children (Haskett, Nears, Sabourin Ward, & McPherson, 2006), and this variability may influence resilience among maltreated children (Herrenkohl EC, Herrenkohl RC, & Egolf, 1994).

Another likely and important predictor of resilience is oxytocin (Feder et al., 2009), given it is a neuropeptide that plays a key role in social behavior and attachment (Bartz, Zaki, Bolger, & Ochsner, 2011; Guastella & MacLeod, 2012; Kumsta & Heinrichs, 2012; Olff, 2012; Olff et al., 2013). For example, oxytocin may play a role in posttrauma resilience by decreasing the physiological stress response and contributing to social connection (Olff, 2012). Recent studies have investigated the relationship of the oxytocin receptor gene with behavioral variables as well as associated biomarkers. One variant of this gene, the OXTR rs53576 polymorphism, has been associated with a range of psychosocial variables (Bradley et al., 2011; Cicchetti & Rogosch, 2012; Costa et al., 2009; Lucht et al., 2009). In general, these data show an allele-load-dependent relationship with adaptive social functioning. Specifically, studies have found that individuals with either one or more copies of the OXTR rs53576 G allele show more adaptive social functioning, whereas those with one or more copies of the minor allele (A) show less adaptive social functioning. Although the genetic function of the rs53576 SNP is not known, a recent study suggests that it may impact hypothalamic–limbic structure and function (Tost et al., 2010).

Not all of the research on OXTR rs53576 has been consistent with the idea that the G allele carriers show more prosocial or other resilience-associated behaviors (Cornelis et al., 2012). One explanation for this variability is that oxytocin may serve the function of increasing the salience or level of responsiveness to social context (Bartz et al., 2011; Kumsta & Heinrichs, 2012). From this perspective, variation in outcome may result from both differential levels of attention and reactivity to social context and the differences in the actual and perceived nature of social environments. Similarly, although most psychological G×E studies focus on genetic variations that confer risk in the context of adverse environmental exposures (e.g., childhood abuse), an alternate perspective suggests that that the inherited phenotype is not “risk” but rather the degree of individual sensitivity to environmental context (plasticity) in response to environmental factors. This would indicate that just as individuals who carry genetic variations associated with environmental sensitivity and plasticity are more vulnerable when exposed to detrimental social/environmental forces, the same individuals are more resilient when exposed to enriching and supportive social/environmental forces (Belsky et al., 2009).

This study seeks to extend the current research in three ways. First, using data from a sample with high levels of adult and childhood trauma exposure, we examined whether positive factors in the childhood family environment (CFE) contributed to adult resilient coping and positive affect, even after accounting for lifetime trauma exposure, including childhood maltreatment. Second, we examined whether OXTR rs53576 genotype predicted the level of adult resilient coping and positive affect. Third, we examined whether the relationship between positive factors in the CFE, adult resilient coping, and positive affect varied across OXTR rs53576 genotype.

## Method

### Procedure

Participants were recruited from the waiting areas of the General Medical and Obstetric/Gynecological Clinics at a publicly funded, non-profit healthcare system that serves a low-income population in Atlanta, Georgia. Eligibility included the ability to give informed consent. Written informed consent was obtained for all participants. Data were collected as part of a larger, NIH-funded, Grady Trauma Project which studies risk and resilience factors following trauma exposure (Binder et al., 2008; Bradley et al., 2008; Gillespie et al., 2009). Participants completed a battery of self-report measures assessing trauma history, childhood abuse, resilient coping, positive affect, socio-demographic characteristics, and other factors. All questions were read aloud to participants by trained interviewers. Across the course of this research, approximately 60% of potential participants approached agreed to participate in the study (Gillespie et al., 2009). Participants were compensated $15 for their time. The presented data were collected between 2005 and 2012.

### Participants

The sample included 971 African American, adult study participants. The number of participants for individual analyses varied as some of the participants did not complete all of the measures.

### Assessment

#### Childhood maltreatment

Childhood maltreatment was assessed with a 28-item self-report Childhood Trauma Questionnaire (CTQ). For this sample, the CTQ had a standardized alpha coefficient of 0.94. The CTQ assesses sexual, physical, and emotional abuse, and emotional and physical neglect. Following Bernstein and Fink's suggested cut-off scores (Bernstein et al., 2003), we classified each type of abuse into one of two categories: none/mild or moderate/severe. Each participant was then given an index for childhood maltreatment, which ranged from 0 to 5 for the number of types of reported moderate/severe childhood maltreatment.

#### Other trauma exposure

Other trauma exposure was assessed using the Traumatic Events Inventory (TEI; Schwartz, Bradley, Sexton, Sherry, & Ressler, 2005), a self-report screening instrument for lifetime history of traumatic events. Consistent with prior reports (Binder et al., 2008; Bradley et al., 2008; Gillespie et al., 2009), we summed the number of traumatic experiences to create a lifetime trauma exposure score.

#### Posttraumatic stress disorder

Symptoms of posttraumatic stress disorder (PTSD) were assessed using the modified PTSD symptom scale (MPSS), a psychometrically sound, 17-item self-report measure assessing PTSD symptoms (Coffey, Dansky, Falsetti, Saladin, & Brady, 1998). For this sample, the MPSS had a standardized alpha coefficient of 0.92.

#### Childhood family environment

CFE was assessed with a 10-item inventory labeled the CFE questionnaire. Two of these items assessed warmth and stability of the CFE and were taken from the self-report Clinical Data Form (DeFife, Drill, Nakash, & Westen, 2010). For the item that assessed warmth, those reporting having had a “warm and nurturing” CFE received a score of 1 and those reporting otherwise (i.e., somewhat warm or cold/unpleasant) received a score of 0. For the item that assessed stability, those reporting having had “a very stable” CFE received a score of 1 and participants reporting otherwise (i.e., stable, somewhat stable, unstable) received a score of 0. The remaining eight items were modified items from two national epidemiological studies, the National Study of Midlife Development in the United States (MIDUS) study (Brim, Ryff, & Kessler, 2004) and the Coronary Artery Risk Development In Young Adults (CARDIA) study (Friedman et al., 1988). These items rated feeling loved, supported and cared for; gesture of physical affection; household organization and management; parental monitoring; feeling understood; being able to discuss worries and confide in others; given time and attention; and parental effort. For each of these eight items, participants who reported the assessed aspect of their CFE as present “most of the time” received a score of 1 and all other responses received a score of 0. We scored items in this instrument dichotomously because we wanted the data to be similar to that used to assess total trauma in our prior research. We also wanted the data to be comparable to data from one of the largest studies examining the relationship of childhood experience and later adult adaptive functioning and wellbeing, the Adverse Childhood Experiences Study (ACES; http://www.cdc.gov/ace/). In these programs of research, total trauma and childhood adversity load is calculated by summing the number of types of traumatic experiences or adversities. Therefore, we decided to score positive family environment in a similar manner. The total score for this 10-item scale ranged from 0 to 10, with the higher score reflecting the more positive the CFE. The first two items (those measuring warmth and stability) have been collected across the full course of our research project whereas the remaining eight items were added later in the study. Therefore, we have a higher number of participants who have responded to the first two items.

#### Resilient coping

Resilient coping was assessed with the 10-item Connor–Davidson Resilience Scale (CD-RISC). The CD-RISC is a self-rated scale with very good psychometric properties (Campbell-Sills & Stein, 2007). Scores range from 0 to 40, with the higher score reflecting greater resilient coping. For this sample, the CD-RISC had a standardized alpha coefficient of 0.88.

#### Positive affect

Positive affect was assessed with the Positive and Negative Affect Scale (PANAS) (Watson, Clark, & Tellegen, 1988). The PANAS is composed of two 10-item scales that measure either positive or negative affect. The total score of the scale measuring positive affect was used in this investigation. For this sample, the PANAS positive affect scale had a standardized alpha coefficient of 0.89.

#### Genotyping

DNA was extracted from saliva collected into Oragene saliva kits (DNAGenotek Inc. Ontario, Canada) using the Agencourt DNAdvance Kit (Beckman Coulter Inc., Brea, CA, USA). The OXTR single-nucleotide polymorphisms, rs53576, were genotyped using Sequenom iPLEX technology (MassARRAY system, Sequenom Inc., San Diego, CA, USA) and Taqman single-nucleotide polymorphism genotyping assays (Life Technologies Corp., Carlsbad, CA, USA). OXTR rs53576 was genotyped using both Taqman (88.0% call rate) and Sequenom (96.7% call rate) with a 28% across method duplication and a discordance rate of 0.1%. Negative controls and within- and across-plate duplicates were used for quality control. Discordant samples were removed prior to analysis. Genotype frequencies were checked for Hardy–Weinberg equilibrium (χ^2^=1.61, *p*=0.21). The minor allele for rs53576, “A,” occurs with a frequency of 0.21 in African Americans.

### Data analyses

All data analyses were conducted using an IBM SPSS Statistics software version 20. We examined the distributions of all key predictor and outcome variables. The PTSD, trauma exposure, and childhood abuse variables were positively skewed, and the resilient coping, positive affect, and family environment variables were negatively skewed. However, the level of skewness (range −0.57–1.07), as well as the level of kurtosis (range −0.37–0.73), fell within acceptable parameters for the sample size (Tabachnick & Fidell, 2000). Pearson product correlations were used to assess the relationship among the key variables.

We conducted two separate hierarchical multiple regression analyses in order to examine whether family environment predicted resilient coping and positive affect after accounting for family environment, trauma-related, and demographic variables. In each of the analyses, demographic variables were entered in the first step, lifetime trauma exposure in the second step, childhood maltreatment in the third step, level of PTSD symptoms in the fourth step, and family environment in the final step. We next examined the relationship of OXTR rs53576 and resilient coping/positive affect. As our initial regression analyses showed a similar relationship between positive family environment and both outcome variables, we standardized our two outcome variables (resilient coping and positive affect) and created one combined outcome variable for this analysis. We first analyzed the data for a main effect of OXTR rs53576. Based on our prior research finding that individuals carrying one or both copies of the rs53576 G allele appeared to be more sensitive to the developmental environment than those carrying both copies of the A allele (Bradley et al., 2011), we coded our genetic variables as a two-level variable (GG and AG vs. AA). We conducted analyses using a generalized linear model predicting the combing resilient coping/positive affect variable with the two-level OXTR rs53576 variable.

We next examined the data for an interaction of OXTR rs53576 and family environment in predicting resilient coping/negative affect. Both phenotype and genotype data for our total sample of 971 were available for the first two items (related to questions about family stability and nurturing environment) of the CFE questionnaire. The additional items were added at a later point in the study and data from all of these participants have not yet been genotyped for OXTR rs53576. Therefore, to conduct these analyses we created a combined childhood family warmth/stability variable. We placed individuals who reported either family warmth or stability or both into one group (*n =* 591) and those individuals who reported neither warmth nor stability in their family environment into the second group (*n =* 380). We used the two-level variable of OXTR rs53576 (GG and AG vs. AA) in this analysis. To confirm this interaction effect, we also conducted the analysis using the non-parametric resampling procedure of bootstrapping (with *n*=5,000 bootstrap resamples).

## Results

The characteristics of the sample including demographics, trauma exposure, and OXTR rs53576 genotype are presented in Table 1. Table 2 presents correlations among positive CFE, childhood maltreatment index, number of lifetime traumatic exposures, PTSD symptoms, resilient coping, and positive affect. As expected, both resilient coping and positive affect were significantly and negatively correlated with childhood maltreatment, other lifetime trauma exposure, and PTSD symptoms, and positively correlated with positive CFE.

**Table 1:** Participant characteristics

Participant variables	
Age M (SD)	34.84 (8.83)
Female (%)	69.7
Household monthly income (%)	
$0–$499	37.5
$500–$999	26.4
$1,000–$1,999	25.1
$2,000 or more	10.0
Moderate or severe childhood abuse (CTQ) (%)	
Physical abuse	20.1
Sexual abuse	27.3
Emotional abuse	18.6
Physical neglect	13.2
Emotional neglect	15.3
Lifetime trauma exposure (TEI) (%)	
Childhood family environment score, M (SD)	6.40 (3.67)
PTSD symptom score, M (SD)	11.92 (12.32)
Resilient coping score, M (SD)	31.61 (7.69)
Positive affect score, M (SD)	36.55 (9.42)
OXTR rs53576 genotype (%)	
GG	62
AG	33
AA	5

CTQ=Childhood Trauma Questionnaire; PTSD, posttraumatic stress disorder; TEI=Traumatic Events Inventory.

**Table 2:** Correlations among positive family environment, childhood maltreatment index, other lifetime trauma exposures, resilient coping, and positive affect (*N*=519)

Variables	1	2	3	4	5	6
Positive family environment	–					
Number of types of child maltreatment	−0.50***	–				
Lifetime trauma exposure	−0.30***	0.45***	–			
PTSD symptoms	−0.38***	0.49***	0.51***	–		
Resilient coping	0.35***	−0.36***	−0.28***	−0.46***	–	
Positive affect	0.34***	−0.29***	−0.20***	−0.36***	0.71***	–

*** *p*<0.001.

PTSD, posttraumatic stress disorder.

We found that CFE significantly predicted resilient coping even after controlling for demographic variables, lifetime trauma exposure, childhood maltreatment, and PTSD symptoms. Similar to resilient coping, we found that CFE significantly predicted positive affect even after controlling for demographic variables, such as lifetime trauma exposure, childhood maltreatment, and PTSD (see Tables 3 and 4 for both predicted variables). Neither total lifetime trauma exposure nor childhood maltreatment remained significant in the final step. However, for both resilient coping and positive affect, PTSD remained significant in the final step, with higher levels of PTSD predicting lower levels of resilient coping and positive affect.

**Table 3:** Regression predicting resilient coping from demographics, lifetime total trauma, childhood abuse, PTSD symptoms and childhood family environment (*N*=510)

Model	*R* ^2^	SE (*R* ^2^)	*F*Δ	*p*	
Sex, age, education, income*	0.06	7.12	8.26	<0.001	
Lifetime total trauma	0.15	6.79	50.84	<0.001	
Childhood maltreatment	0.19	6.62	28.65	<0.001	
PTSD symptoms	0.27	6.31	50.34	<0.001	
Childhood family environment	0.30	6.18	23.05	<0.001	
Coefficients—final step	*b*	SE (*b*)	*t*	*p*	VIF
Sex	−0.51	0.73	−0.70	0.49	1.20
Age	−0.001	0.02	−0.07	0.40	1.11
Education	0.69	0.17	3.99	0.005	1.07
Income	0.59	0.21	2.79	<0.001	1.07
Lifetime total trauma	−0.07	0.12	−0.60	0.55	1.64
Childhood maltreatment	−0.18	0.03	−6.58	0.45	1.87
PTSD	−0.29	0.37	−0.76	<0.001	1.60
Childhood family environment	0.44	0.09	4.80	<0.001	1.54

* The variable for sex was coded as male=0 and female=1. The age variable was continuous. The education variable was an ordinal variable with the following values: 0 ≤ 12th-grade education, 1=12th-grade education, 2=GED, 3=some postsecondary education, 4=technical school graduate, 5=college graduate, 6=graduate school-level education. The income variable was ordinal with the following values: 0 ≤ $250/month, 1=$250–499/month, 2=$500–999/month, 3=$250–499/month, 4=$1,000–1,999/month, 5 ≥ $1,999/month. PTSD, posttraumatic stress disorder; VIF, Variance Inflation Index.

**Table 4:** Regression predicting positive affect from demographics, lifetime total trauma, childhood abuse, PTSD symptoms and childhood family environment (*N*=510)

Model	*R* ^2^	SE (*R* ^2^)	*F*Δ	*p*	
Sex, age, income, education*	0.05	8.25	7.24	<0.001	
Lifetime total trauma	0.09	8.08	22.31	<0.001	
Childhood maltreatment	0.12	7.95	17.82	<0.001	
PTSD symptoms	0.17	7.73	29.62	<0.001	
Childhood family environment	0.24	7.45	39.64	<0.001	
Coefficients—final step	*b*	SE (*b*)	*t*	*p*	VIF
Sex	0.97	0.88	1.09	0.27	1.21
Age	−0.02	0.03	−0.75	0.45	1.12
Education	0.68	0.21	3.25	0.002	1.07
Income	0.68	0.21	3.20	0.001	1.07
Lifetime total trauma	0.08	0.14	0.57	0.57	1.64
Childhood maltreatment	0.20	0.45	0.45	0.65	1.87
PTSD	−0.16	0.03	−0.4.80	<0.001	1.60
Childhood family environment	0.70	0.11	6.30	<0.001	1.54

* The variable for sex was coded as male=0 and female=1. The age variable was continuous. The education variable was an ordinal variable with the following values: 0 ≤ 12th-grade education, 1=12th-grade education, 2=GED, 3=some postsecondary education, 4=technical school graduate, 5=college graduate, 6=graduate school-level education. The income variable was ordinal with the following values: 0 ≤ $250/month, 1=$250–499/month, 2=$500–999/month, 3=$250–499/month, 4=$1,000–1.999/month, 5 ≥ $1,999/month. PTSD, posttraumatic stress disorder; VIF, Variance inflation index.

**Fig. 1:**
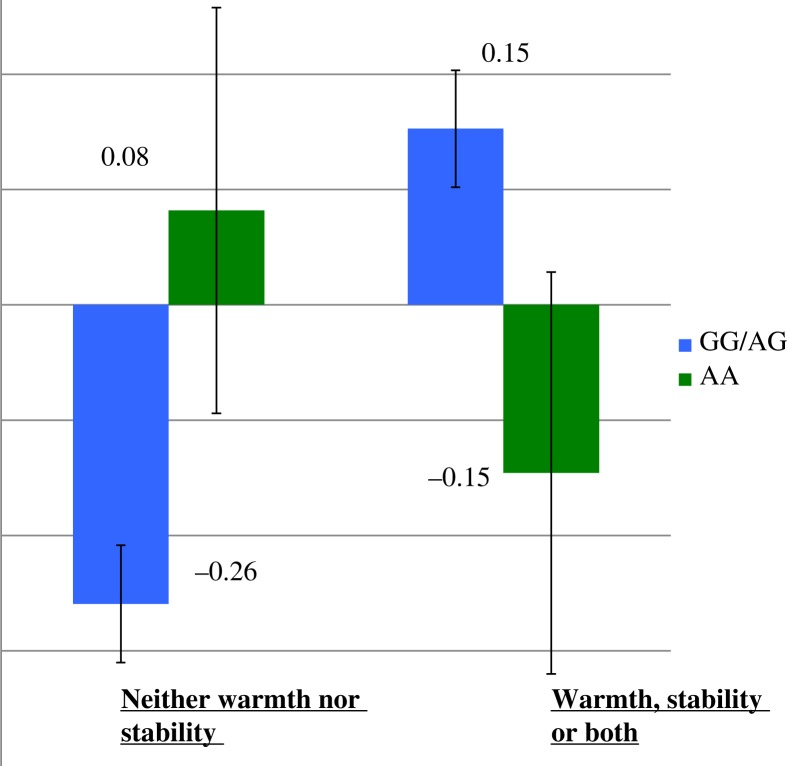
Combined positive affect and resilient coping, OXTR rs53576 genotype and level of family warmth and stability. Note: The combined positive affect and resilient coping data are presented as *z* scores and error bars reflect standard deviation.

We did not find evidence of a main effect for OXTR rs53576 genotype (GG and AG vs. AA) on the combined resilient coping/negative-affect-dependent variable (Wald χ^2^=0.055, *p*=0.97). However, our data did suggest an interaction of OXTR rs53576 genotype and positive family environment in predicting resilient coping/positive affect (Wald χ^2^=5.12, *p =* 0.024). Bootstrapping analysis also showed a significant interaction (*B*= − 0.62, *p*=0.012). *Post-hoc* pairwise comparisons found a significant difference (*p*<0.001) in resilient coping/positive affect between those individuals with the GG and AG genotypes who were raised in a stable or warm family environment (*N*=557, M=0.15, SD=0.92), and those individuals in same group (GG and AG genotypes) not raised in a warm or stable family environment (*N*=363, M=− 0.26, SD=0.96). However, when comparing AA genotype individuals raised in a warm or stable family environment (*N*=34, M=0.15, SD=1.02) to those individuals with the AA genotype (*N*=17) who were not raised in a warm or stable family environment (M*=*0.08, SD=0.73), data analyses did not suggest a significant difference in resilient coping/positive affect (see Fig. 1).

## Discussion

We found that positive affect and resilient coping were both positively correlated with positive CFE and negatively correlated with childhood maltreatment and other traumatic experiences. Furthermore, we found that even after adjusting for childhood maltreatment, other traumatic experiences, and PTSD symptoms, a more positive CFE was associated with higher levels of positive affect as well as resilient coping. These findings highlight the importance of examining protective developmental factors in addition to childhood maltreatment, and suggest that even in the face of adversity, positive aspects of the family environment may contribute to resilience.

In addition, our data suggested that the impact of positive CFE varied across OXTR rs53576 genotype. Consistent with other studies, we found that for individuals with the GG and AG genotype, a more positive CFE was associated with higher levels of positive affect and resilient coping in adulthood. Chen and colleagues (2011) found that individuals with one or two copies of the OXTR rs53576 G allele had lower cortisol levels in response to stress only if they received social support. In the condition without social support, this effect was not found, suggesting that this genetic variation impacts the degree to which social support buffers the effects of stress. Similarly, research on the relationship of inter-parental conflict with maternal sensitivity found that individuals with the OXTR rs53576 GG allele showed higher levels of maternal sensitivity than AA and AG individuals only if inter-parental conflict was low (Sturge-Apple, Cicchetti, Davies, & Suor, 2012). The data presented here add to our understanding of factors predicting resilience by extending it to include two measures of adult resilience and genetic variation in a sample of African American adults with high rates of child maltreatment and adult trauma exposure. This research suggests that these models should be expanded to take into account the role of positive family environment in addition to childhood maltreatment.

The results of this study should be interpreted in the context of its several limitations. First, this was a cross-sectional study. Future research should examine family environment and trauma exposure as predictors of risk and resilience over the course of childhood and adolescent development. We have recently begun a study examining risk and resilience among children and their mothers in this population (Jovanovic et al., 2012). Second, our assessment of CFE, childhood maltreatment, other traumatic experiences, resilient coping, and positive affect was based on self-report questionnaires, which are susceptible to recall bias. For example, it may be that a resilient coping style and positive affect impact the way in which an individual remembers their CFE. Third, for the genetic analyses, the sample size was small. In particular, the AA group was very small in this sample. Replication of the results with a larger sample size is needed. In addition, this research was conducted in a sample that was homogenous with respect to income and race, and the results may not generalize to other populations. Fourth, we only had data on two of our questions about family environment for the full sample. Therefore, we conducted our G×E analyses using only these variables. We added additional items to the CFE during the course of our research to assess positive family environment more comprehensively than has been done in other research on trauma-related risk and resilience. We hope to eventually conduct more comprehensive analyses of the G×E data that includes all items in this scale. Finally, resilience is a broad term that is used to describe a number of ideas. The way we assessed resilience in this article reflects only one perspective on resilience and more research using additional approaches to resilience is needed.

Despite these limitations, this study presents important empirical findings based on an ethnic minority population with high rates of trauma exposure, a significant associated risk for adverse outcomes. Our data suggest that even in the face of adversity, positive aspects of the family environment may contribute to resilience. Understanding factors that contribute to resilience have the potential to inform broader public health policies and practices for this vulnerable population in particular, and the broader population in general. We found that the impact of positive CFE varied across OXTR rs53576 genotype. To our knowledge, this is the first study examining the relationships between positive CFE, OXTRrs53576, resilient coping, and positive affect. Data from this study also point to the importance of examining a full range (positive and negative) of environmental variables, as well as phenotype variables, when studying the relationship of childhood environment to adult adaptive functioning.
